# Raman Spectroscopy–Based Quantitative Analysis of Fatty Acid Compositions of Lipid Droplets in Live Cells

**DOI:** 10.1021/acs.analchem.5c04227

**Published:** 2026-03-09

**Authors:** Pradjna N. Paramitha, Keita Iwasaki, Bibin B. Andriana, Yurika Otoki, Ibuki Kusumoto, Yukihiro Ozaki, Kiyotaka Nakagawa, Hidetoshi Sato

**Affiliations:** † Graduate School of Science and Technology, Kwansei Gakuin University, 1 Gakuen Uegahara, Sanda, Hyogo 669-1330, Japan; ‡ School of Biological and Environmental Sciences, Kwansei Gakuin University, 1 Gakuen Uegahara, Sanda, Hyogo 669-1330, Japan; § Food Function Analysis Laboratory, Graduate School of Agricultural Science, 13101 Tohoku University, Sendai, Miyagi 980-8572, Japan

## Abstract

The quantitative analysis of fatty acids (FAs) and their acyl group compositions in triacylglycerols (TAGs) has become one of the main areas of interest for understanding the metabolism and function of fats in the body. Although Raman spectroscopy and chemometric-based analytical methods have previously been applied for directly and nondestructively analyzing fats, fat samples are difficult to quantitatively analyze because an appropriate analytical model must be constructed based on a known calibration data set before applying the model to unknown samples. Therefore, we developed a technique to construct calibration models for fatty acyl groups using simulated TAG spectra generated from fatty acid methyl esters (FAMEs) spectra. Because of the vast diversity of TAGs and high prices of commercial pure reagents, the preparation of accurate concentrations of TAGs for training models would be very difficult and costly. Classical and nonnegative least-squares regressions (CLSR and NNLSR, respectively), which do not require calibration modeling, were compared with analyses using partial least-squares regression (PLSR). A comparative analysis revealed that the combination of PLSR modeling with simulated calibration data sets produced the most accurate predictions. The PLSR models were evaluated using edible oils and, compared to the results obtained using gas chromatography, the models reasonably approximated the fatty acyl group compositions in the fat samples. Then, the models were applied to estimate fatty acyl group compositions in live, single adipocytes. Although the models’ accuracies were limited, they nondestructively estimated the fatty acyl group compositions of LDs in live cells.

## Graphical abstract

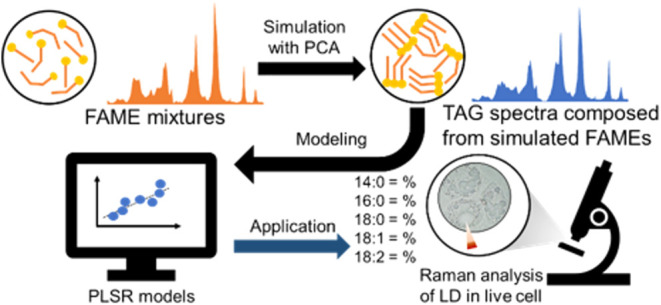

## Introduction

Fat is a major component of both foods and human and animal bodies. When fat is ingested, it is decomposed to fatty acids (FAs), absorbed in the intestines, and packaged into chylomicrons (CMs),^1,2^ which are sent to the lymphatic system, enter the bloodstream, and are delivered to cells for generating energy.^3,4^ Any fat that has not been used for energy accumulates as triacylglycerols (TAGs) in adipocytes.^5,6^ In biological samples, fats mostly comprise TAGs, where three FAs are connected to one glycerol molecule via ester bonds. Additionally, some FAs may be decomposed and used to synthesize different FAs.^7,8^ To fully understand FA metabolism, further knowledge is required about the consumption and synthesis of FAs in cells, as well as FAs cycling between organs in the body.

Obesity is often used as an indicator to estimate human health conditions. To assess obesity, body fat is commonly analyzed as the “total fat,” without further analyzing the fatty acyl group composition. However, over the last few decades, several studies have suggested that some diseases are correlated with the excess dietary consumption of certain FAs and their subsequent accumulation in body fat,^9–12^ implying the importance of the quantitative analysis of individual FAs in biological samples both for studying fat metabolism–related diseases and practical health-control applications. Therefore, to quantify FA metabolism in the human body for health control and medical analyses, we used Raman spectroscopy, a noninvasive optical measurement method, to develop instrumental techniques, including fiber optic Raman probes for subsurface sensing, and chemometric-based quantitative analytical techniques for monitoring live cells and tissues.^13,14^

Meksiarun et al. used a ball lens top/hollow optical fiber Raman probe (BHRP) to noninvasively analyze the concentrations of specific fatty acyl groups in the subcutaneous fat of live hamsters.^13^ Their results suggested that when the hamsters consumed trilinolein or tricaprin, the accumulation rate of linoleic acid group was significantly greater than that of the decanoic (capric) acid group. In addition, Raman spectroscopy has been applied for monitoring fat accumulation in live cells.^14^ HepG2 cells, a liver model cell line, induced cell death when it was cultured with high concentrations of linoleic acid. Such contrasting responses to linoleic acid in adipocytes and hepatocytes serve as an example highlighting the importance of quantitative analysis of fatty acyl composition in live tissues and cells. Because of their noninvasiveness and nondestructiveness, Raman spectroscopy–based techniques are highly valuable for health monitoring and various medical applications.^15,16^ Furthermore, for understanding fat metabolism, the quantitative analysis of fatty acyl groups, which may be important health indicators, in certain cells and tissues is crucial.

Fats are generally analyzed qualitatively by chromatographic methods. Because fats include various fatty acyl groups possessing similar structures and different carbon chain lengths, numbers of double bonds, and *cis*- or *trans*-conformations, detailed fat analyses include relatively complex sample preparations and data analyses.^17^ Prior to the application of chromatographic methods for quantitatively analyzing fatty acyl group compositions, fats must first be extracted, purified, and derivatized to release free FA derivatives (e.g., via transmethylation). Because the polarities and solubilities of various fats are different, extraction solvents must be carefully selected to maximize lipid extraction from samples.^18^ Nonetheless, because of their destructiveness, chromatography-based methods are infeasible for analyzing fats in live cells and tissues.

Despite numerous reports on Raman spectroscopy–based fat analysis, many previously published studies have relied on methods that describe the saturation degree.^19–21^ Therefore, a notable gap between conventional Raman spectroscopy–based analytical methods and the quantitative analysis of fatty acyl groups in TAGs remains in the literature. In the Raman spectroscopy–based analysis of FAs or fatty acyl groups, spectral overlap, attributed to similar structures, is a major challenge. To obtain values from a spectrum, a calibration curve is necessary. However, simple analytical methods, such as univariate analysis, are not suitable for application to the complex spectral data sets. Multivariate analysis, also known as chemometrics, is well-suited for obtaining calibration curves—referred to as calibration model or analytical model in multivariate analysis—for such complex Raman spectra.^22,23^ In this study, fats and FAs were quantitatively analyzed using the following chemometric methods: classical,^23,24^ nonnegative,^25^ and partial least-squares regressions^26^ (CLSR, NNLSR, PLSR, respectively), and the results were subsequently compared.

Another challenge is the high cost and labor involved in constructing analytical models for TAG reagents. Even if good Raman spectra of fats can be obtained, quantitative information cannot be extracted without an analytical model. Meksiarun et al. used resected adipose tissues of hamsters as training samples and constructed quantitative analytical models of the fatty acyl groups using their spectra and the concentrations determined by gas chromatography (GC).^13^ However, this method cannot be applied to individual cells, even in humans. Therefore, the analytical models must be constructed using artificially prepared training samples. In preparing training and test samples, the cost of pure TAG reagents, which are sometimes commercially available, has often been prohibitive. Numerous samples containing the exact concentrations of specific molecules of interest must be prepared. Moreover, in principle, training data sets must cover the possible data dispersion of samples. Because TAG molecules can comprise three different fatty acyl groups, approximately *n* ^3^ TAG types are possible for *n* fatty acyl group types. However, because of the huge number of possible combinations of three fatty acyl groups in TAG molecules, the preparation of pure reagents for all TAGs is experimentally quite difficult. Furthermore, in numerous mixed training and test samples, TAG reagent concentrations must be strictly controlled by weighing the reagents.

In this study, we quantified the fatty acyl group compositions of individual lipid droplets (LDs) in live adipocytes. To understand the relationships among the synthesis, modification, and consumption of individual fatty acyl groups, the monitoring of changes in fatty acyl groups’ compositions is useful and provides deep insight into fat metabolism in live adipocytes. LDs are rich in TAGs, which form highly dense liquid aggregates. Because TAGs are hydrophobic and most other biological molecules in the cytoplasm are usually hydrophilic, the latter are excluded. In LDs, sample variability is attributed to TAG-derived fatty acyl groups, which differ based on their hydrocarbon chain lengths and numbers of double bonds. To construct a model for quantitatively analyzing specific fatty acyl groups, although TAG-based training samples initially had to be prepared, this was very difficult for the reasons we have already described. Hence, we develop a technique to generate a calibration data set for nonexistent samples, such as TAG mixtures, from a data set of existing reagents possessing similar molecular structures by simulation and evaluated the feasibility of the proposed technique.

## Materials and Methods

### Materials

Methyl myristate (MAm, 14:0, >99.5%), methyl palmitate (PAm, 16:0, >99.5%), methyl stearate (SAm, 18:0, >99.5%), methyl oleate (OAm, 18:1, >99.0%), and methyl linoleate (LAm, 18:2, >98.0%) were purchased from Tokyo Chemical Industry (Tokyo, Japan), while trimyristin (≥99.0%), triolein (≥99.0%), and trilinolein (≥97.5%) were obtained from Sigma–Aldrich (St. Louis, MO, USA). Tripalmitin (>98.0%), tristearin (>98.0%), butylhydroxytoluene (BHT)—used as solvent stabilizers for fat extraction and transmethylation—fetal bovine serum (FBS), dexamethasone, isobutylmethylxanthine (IBMX), and insulin were acquired from Fuji Film Wako Pure Chemical Corporation (Osaka, Japan). These reagents were freshly purchased and used without any further purification for the experiments. Because the reagents’ purities were sufficiently high, we ignored the reagents’ impurities in subsequent calculations. We used FAME standard mixes GLC-461A, GLC-68C, and GLC-68D (Nu-Chek Prep, Elysian, MN, USA) as references for identifying FAMEs via GC coupled with flame ionization detection (FID). A mouse preadipocyte cell line, MC3T3-G2/PA6 (RCB1127), was obtained from the RIKEN Cell Bank (RIKEN Bioresource Research Center, Tsukuba, Japan). The cell culture medium and supplements (α-minimum essential medium (MEM) and penicillin/streptomycin, respectively) were purchased from Thermo Fisher Scientific, Inc. (Waltham, MA, USA). Quartz-bottomed dishes were purchased from Fine Plus International Ltd. (Kyoto, Japan) for measuring the Raman spectra of live cell cultures. For solid-phase extraction (SPE), a silica column (SI-1, 100 mg, 1 mL, Strata) was obtained from Phenomenex (CA, USA). Olive and sesame oils were edible grades and purchased at a local market in Hyogo, Japan.

### FAME Samples

Because fat mixtures were prepared by mixing the FAMEs (MAm, PAm, SAm, OAm, and LAm) at various concentrations ranging from 0% to 100% (mol/mol), fatty acyl group compositions were expressed as mole percentages. For each FAME type, five analytical models were constructed in PLSR analysis, and each model was evaluated using test samples. For constructing and evaluating the models, we prepared 165 training and 20 test FAME mixture samples.

### Cell Cultures

A mouse preadipocyte cell line (MC3T3-G2/PA6) was cultured in a standard medium, i.e., α-MEM, supplemented with 10% FBS and 1% penicillin–streptomycin. For measuring Raman spectra, 2 × 10^5^ preadipocytes were seeded in a 35 mm diameter quartz-bottomed dish. Concurrently, for extracting fats for GC analysis, 1.6 × 10^6^ preadipocytes were seeded in a 100 mm diameter cell culture dish. To induce the differentiation of preadipocytes into matured adipocytes,^27^ the culture medium was replaced with a standard medium (additionally supplemented with 5 × 10^4^ M IBMX, 25 nM dexamethasone, and 5 μM insulin). The medium was replaced with new one every 3 days. According to the visual observation, their differentiation was finished within 6 days. The cells were cultured for an additional 6 days and then processed as follows: Raman spectra were measured for the adipocytes in quartz-bottomed dishes. For GC analysis, cells were harvested using a cell scraper, diluted in 1 mL of phosphate-buffered saline (PBS(−)), and frozen at −80 °C for fat extraction. To ensure independent experimental reproducibility, cell samples were prepared five times with the same procedure for both Raman spectrum measurements and GC analyses.

### Measurement of Raman Spectra for FAME and Edible Oil Samples

A Raman microscope system comprising a 785 nm wavelength diode laser (Toptica Photonics, Munich, Germany) coupled with a single polychromatic Raman spectrometer (F4.2, 320 mm focal length, 750 nm blazed 600 lines mm^–1^ grating; Photon Design Co., Ltd., Tokyo, Japan), a charge-coupled device detector (DU420-BRDD; Andor Technology Co., Ltd., Northern Ireland), and an objective lens (×20 magnification, numerical aperture (NA) = 0.40; Olympus, Tokyo, Japan) was constructed in house. For each FAME mixture, measurements were repeated three times. At the sampling point, the laser power was 50 mW, and the acquisition time for measurement was 30 s (10 s × 3). Additionally, under the same conditions, Raman spectra were recorded for olive and sesame oils and TAGs containing the same acyl groups in liquid form (trimyristin, tripalmitin, tristearin, triolein, and trilinolein).

### Raman Spectrum Measurements of Cultured Live Mouse Adipocytes

The in-house-constructed confocal Raman microscope system equipped with a CO_2_ incubator (5% CO_2_, 37 °C) was used for measuring the Raman spectra of live adipocytes. Although the Raman microscope was the same as that used for measuring the FAME samples, it was equipped with a water-immersed objective lens (×60, NA = 1.2; Olympus, Tokyo, Japan) instead of the objective lens used for the FAME measurements. The laser spot was approximately 0.8 μm laterally × 1.1 μm deep. The spectra were measured in 30 s (10 s × 3) by focusing on LDs in adipocytes at a laser power of 50 mW at the sampling point. After laser irradiation at 785 nm and 50 mW for 30 s, no live adipocyte showed any visual signs of irregular reactions. For each cell culture, spectra were randomly recorded for 30 LDs. To ensure experimental reproducibility, the whole experimental procedure was repeated five times.

### Raman Data Analysis—Preprocessing

Before chemometric analysis, raw Raman spectra were preprocessed by subtracting background noise spectra arising from the quartz glass and culture medium (for live cell measurements). A weak baseline undulation, attributed to fluorescence and stray light, was corrected using polynomial line fitting. For each spectrum, the intensity was normalized using an internal standard, where a band corresponding to the ester C=O stretching mode at 1770–1720 cm^–1^ was used.

### Raman Data Analysis—FAME Chemometrics

The samples’ spectra were further analyzed by both CLSR and NNLSR in Python 3.11 using the linear model module in scikit-learn.^28^ For CLSR and NNLSR, the root-mean-square errors (RMSEs) of the prediction models (i.e., RMSEP values) were calculated based on the 20 test samples. PLSR analysis was conducted using commercial chemometric software (Unscrambler, CAMO, Bedford, MA, USA). The PLSR model was constructed based on 165 FAME mixture samples for training, and the model’s prediction accuracy was subsequently evaluated based on 20 independent test samples. The calibration model’s RMSE (RMSEC) was calculated based on the training data, and RMSEP was calculated based on the 20 test samples.

### Raman Data Analysis—PLSR Analysis for TAGs

To predict the fatty acyl group compositions of TAGs, PLSR models were constructed. FAME mixture spectra were converted to simulated TAG mixture spectra. We used principal component analysis (PCA) to determine the differences between FAME and TAG spectra and coefficients, indicated by loadings and scores. FAMEs comprising a specific FA were compared to TAGs containing the three acyl groups of that FA.

The following formula was used to determine the spectral variance (Δ*S*_D*i*_)­

1 $${\Delta S}_{Di}=\left({\mathrm{Sc}}_{\mathrm{TAG}i}-{\mathrm{Sc}}_{\mathrm{FAME}i}\right)\cdot L_{\mathrm{PC}1i}$$

where *i* represents a specific fatty acyl group, namely myristic acid (MA), palmitic acid (PA), stearic acid (SA), oleic acid (OA), or linoleic acid (LA); Δ*S*_D*i*_ is the spectral difference between a TAG and its corresponding FAME; *Sc*_TAG*i*_ and *Sc*_FAME*i*_ are the PC1 scores for the TAG and FAME, respectively; and *L*_PC1*i*_ is the PC1 loading. The simulated spectrum of the TAG mixture (*S*_TAG*x*_) comprises the spectrum of the corresponding FAME mixture (*S*_FAMEx_) and Δ*S*_*D*i_ for the fatty acyl groups as follows

2 $$S_{\mathrm{TAG}x}=S_{\mathrm{FAME}x}+{\Delta S}_{D\mathrm{MA}}C_{\mathrm{MAm}}+{\Delta S}_{D\mathrm{PA}}C_{\mathrm{PAm}}+{\Delta S}_{D\mathrm{SA}}C_{\mathrm{SAm}}+{\Delta S}_{D\mathrm{OA}}C_{\mathrm{OAm}}+{\Delta S}_{D\mathrm{LA}}C_{\mathrm{LAm}}$$

where *C*_i_ is the concentration of each FAME containing the specific fatty acyl group (*i*) in the mixture.

To predict fatty acyl group compositions, PLSR models were trained using a data set comprising 165 simulated TAG spectra. The PLSR models were evaluated using 20 simulated TAG spectra and edible oils, i.e., olive and sesame oil. Additionally, hierarchical clustering analysis (HCA, i.e., Euclidean-distance-based median linkage clustering) was conducted by applying Unscrambler to the spectra of 50 LDs in multiple cells to investigate the heterogeneity of the fatty acyl group composition in each LD and among cells.

### GC Analysis

Detailed procedures for fat extraction and substitution modification for GC analysis are described in Supporting Information. The FAMEs extracted from adipocytes and edible oils were analyzed using GC equipped with a FID. For analyzing adipocytes and edible oils, we employed GC-4000 (GL Sciences Inc., Tokyo, Japan) and GC-2010 Plus (Shimadzu, Kyoto, Japan), which were equipped with a DB-225 column (30 m × 0.25 mm diameter × 0.25 μm thick film; Agilent Technologies, Santa Clara, CA, USA). Helium (He) constantly flowing at 100 kPa was used for the carrier gas. The injector and detector temperatures were set at 220 and 250 °C, respectively. The oven temperature was initially set at 140 °C, subsequently raised to 180 and 220 °C at 8 and 3 °C min^–1^, respectively, and maintained at 220 °C for 12 min.^33^ For each analysis, a 5 μL sample of FAMEs in hexane was injected into the instrument. FAMEs were identified by comparing their retention times with those of standard FAME mixtures. FAME concentrations were determined by comparing FAME peak areas to the total peak area in the GC chromatogram.

## Results and Discussion

### Development of the Model for Quantitatively Analyzing Fatty Acyl Groups in Fats

Initially, although we were supposed to use pure FAs instead of TAGs to estimate the fatty acyl group compositions of fat samples, we immediately encountered several difficulties in preparing training samples comprising pure FAs. One of the difficulties was that homogeneous FA mixtures were impossible to prepare. For example, in human cells, major FAs comprising TAGs are MA, PA, SA, OA, and LA, which melting points are 327.05, 334.95, 341.95, 286.55, and 266.25 K, respectively. Although these FAs are either solids or liquids at room temperature, their corresponding TAGs are liquids at body temperature. High-melting-point FAs crystallized and could not be homogenized at room temperature. Moreover, because crystal/solid FAs and TAGs generated inconsistent Raman spectra exhibiting substantially different band ratios because of multiple crystal orientations and polarizations,^34^ solid FA mixtures could not be applied as training samples to construct analytical models. Although we also attempted to prepare solutions of mixed FAs, we could not find a suitable solvent, in which all the FAs were highly soluble yet which spectral bands did not overlap those of the FAs. Furthermore, because of molecular interactions between FAs, the spectra of pure FAs substantially changed, hindering the mimicking of TAG spectra. A band attributed to the C=O stretching mode, originally expected to be near 1735 cm^–1^, was substantially red-shifted and overlapped with a band near 1660 cm^–1^, because pure FAs formed a ring dimer via hydrogen bonds between −C=O and −OH groups in the carboxylic acid.

Therefore, to construct the analytical model, we employed FAMEs instead of TAGs or FAs. Because FAMEs have relatively low melting points, the preparation of a homogeneous mixture is easy at a lower temperature (∼313.15 K). In human cells, TAGs mostly comprise fatty acyl groups on long-length hydrocarbon chains containing 14–18 carbon atoms and either one or two double bonds.^35^ To predict the fatty acyl group compositions of fats, MAm (14:0), PAm (16:0), SAm (18:0), OAm (18:1), and LAm (18:2) were used to prepare the samples for training the analytical models. The Raman spectra and molecular structures of the FAMEs are shown in Figure 1A. The band at 1740 cm^–1^ was assigned to the ester group’s C=O stretching mode.^36^ Because the FAMEs contained a single C=O group, their spectra were normalized based on the area intensity of the C=O band in the region 1770–1720 cm^–1^. Moreover, the C=O group was spatially isolated from structural variations in the alkyl chain. The band at 1439 cm^–1^ and its shoulder were assigned to CH_2_/CH_3_ bending vibrations.^34,37^ Among the saturated FAMEs, SAm generated the most intense band at 1439 cm^–1^ (Figure 1A­(c)), which weakened with decreasing chain length for PAm (16:0) and MAm (14:0). The bands near 1650 and 1260 cm^–1^ were attributed to the C=C stretching and C=C–H bending modes of double bonds^34,36^ and were missing from the spectra of the saturated FAMEs, among which although the spectral features above 1200 cm^–1^ varied, the band shapes and peak wavenumbers were very similar for corresponding bands. Hence, the concentrations of individual FAMEs in the mixture were difficult to estimate because their corresponding spectral bands overlapped. In contrast, the bands below 1100 cm^–1^, especially those near 850 cm^–1^, showed more characteristic features. Thus, these bands were more useful for distinguishing FAMEs, particularly saturated FAMEs. It should be noted that the bands in the region of 838–890 cm^–1^ have significant contributions from the C–O–CH_3_ group in the ester bonding of FAMEs. TAGs will exhibit different spectral characteristics in this region because their acyl groups have an ester bonding with the glycerol group. The Raman bands assigned to the fats are listed in Supporting Information (Table S1).

**Figure 1:**
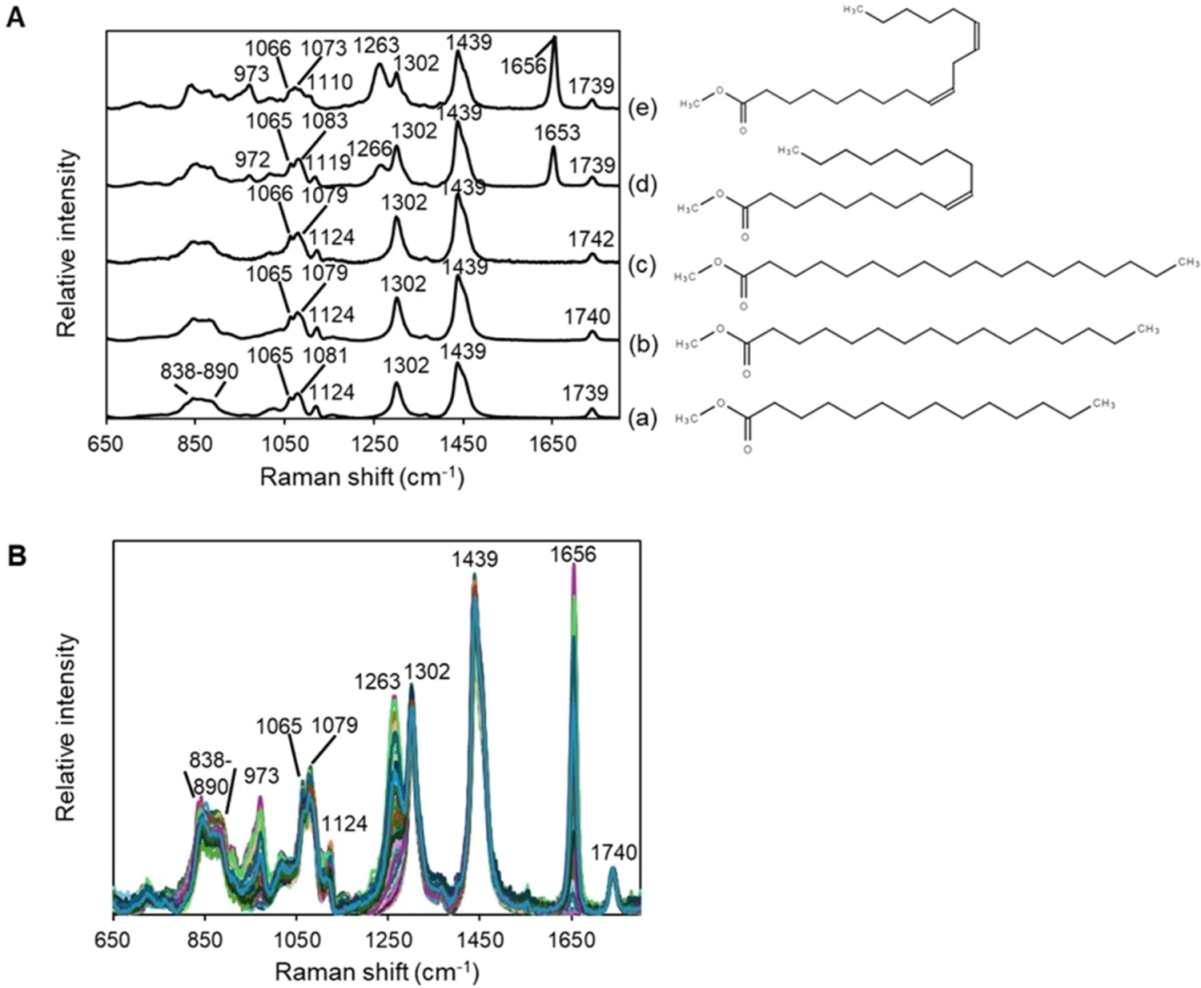
(A) Raman spectra and chemical structures of pure FAMEs and (B) Raman spectra of 165 training samples comprising (a) MAm, (b) PAm, (c) SAm, (d) OAm, and (e) Lam FAMEs.

Figure 1B shows the 165 training data sets comprising Raman spectra generated for FAME mixtures. For each FAME, we compared the concentrations quantitatively estimated using CLSR, NNLSR, and PLSR. Neither CLSR nor NNLSR required training samples for constructing analytical models; instead, they required the pure spectra of all the components comprising the mixture. Therefore, because they do not incur any training-sample-related costs, CLSR and NNLSR are advantageous for analyses where training samples are unavailable. In contrast, PLSR requires the construction of an analytical (prediction) model prior to analyzing unknown data, and although PLSR does not require the spectra of the pure constituent components, it does require the construction of a separate model for each target substance. These analytical models were validated using the 20 independent test samples of FAME mixtures. (Detailed data processing information is described in the Supporting Information.)

The evaluation results obtained using CLSR, NNLSR, and PLSR are shown in Table S2 in the Supporting Information. To evaluate the accuracies of the analytical methods, RMSEC and RMSEP values were calculated. The RMSEP value is obtained using 20 independent test samples and the following equation

$$\mathrm{RMSEP}=\sqrt{\frac{\sum_{i=1}^{n}{\left({ŷ}_{i}-y_{i}\right)}^{2}}{n}}$$

Here, “*ŷ*_*i*_” represents the predicted FAME concentration in mixture “*i*” as determined by PLSR analysis, and “*y*” is the actual FAME concentration in the mixture. It suggests the possible errors in the true values, which in this case are the concentrations of each FAME in the mixture. Among the analytical models, PLSR showed the highest prediction accuracy. PLSR possessed 0.97 for the lowest *R* ^2^ value and 6.9 for the highest RMSEP value among the five models. In contrast, CLSR and NNLSR possessed 65.6 and 32.5, respectively, for the highest RMSEP values. Although CLSR and NNLSR simultaneously predicted the compositions of all the targeted substances, the corresponding large errors indicated that these models were insufficient for the analysis. PLSR was specifically designed to model the covariance between predictor and response matrices. This approach effectively separates predictive information from noise and random error, enabling the robust modeling of independent test samples. For OAm and the model constructed based on the spectral regions 1700–1400 and 1280–650 cm^–1^, the RMSEP value was 3.9, and for LAm and the model constructed based on the spectral region 1700–650 cm^–1^, the RMSEP value was 2.4. For these models, the prediction accuracies were slightly higher than those of the saturated FAME models because the bands attributed to double bonds in fatty acyl groups were substantially different. For MAm and the model constructed based on the spectral region 1700–650 cm^–1^, the RMSEP value was 3.9, and for PAm and SAm and the models constructed based on the spectral region 1500–650 cm^–1^, the RMSEP values were 6.4 and 4.7.

### Construction of the Model for Quantitatively Analyzing TAG Samples

We directly applied the FAME-spectrum-based PLSR models for predicting the fatty acyl group compositions of TAGs, i.e., edible oils (Table S3), which substantially reduced the models’ prediction accuracies, suggesting that despite the similar structures of FAMEs’ and TAGs’ fatty acyl groups, subtle variations in FAME and TAG spectral features impacted the models’ prediction accuracies. To overcome these limitations, we used simulated spectra constructed based on similar substances to construct models for analyzing nonexistent substances. The Raman spectra of pure FAMEs and TAGs comprising (a) MA, (b) PA, (c) SA, (d) OA, and (e) LA are compared in Figure 2A. Although these spectra were highly similar, they were slightly different. These spectral differences arose from the distinct FAME and TAG molecular structures. In FAMEs or TAGs, one or three FA molecule(s) was (were) esterified to a methanol or a glycerol moiety, respectively. These structural variations primarily manifested as changes in band positions and intensities at 1740 cm^–1^ and in the 1700–1600, 1500–1400, and 900–800 cm^–1^ regions (Figure 2B). The band assigned to the C=O stretching mode of ester groups in TAGs was slightly blueshifted and broader than the counterpart band assigned to the C=O stretching mode of ester groups in FAMEs, which may be because TAG molecules contain multiple C=O bonds. The bands varied in the 1700–1600 cm^–1^ region, only in the spectra of the unsaturated fatty acyl groups, suggesting that in TAGs, interactions among the three fatty acyl groups slightly altered the structures around the double bonds in the hydrocarbon chains.^38^ In the 1500–1400 cm^–1^ region, spectral band variations were correlated with different CH_3_ and CH_2_ group ratios relative to the C=O group in FAMEs and TAGs. The bands at approximately 900–800 cm^–1^ were possibly attributed to the hydrocarbon chains’ C–C and ester bond’s C–O–C stretching modes.^36^ Because of these differences between the FAMEs’ and TAGs’ spectra, TAG samples poorly fit the analytical model, producing poor prediction accuracies when models constructed based on FAME spectra were used to estimate the fatty acyl group compositions of TAG-containing samples. Although these spectral differences appear to be minor, PLSR models constructed based on FAME data sets must be modified prior to estimating the fatty acyl group compositions of TAGs.

**Figure 2:**
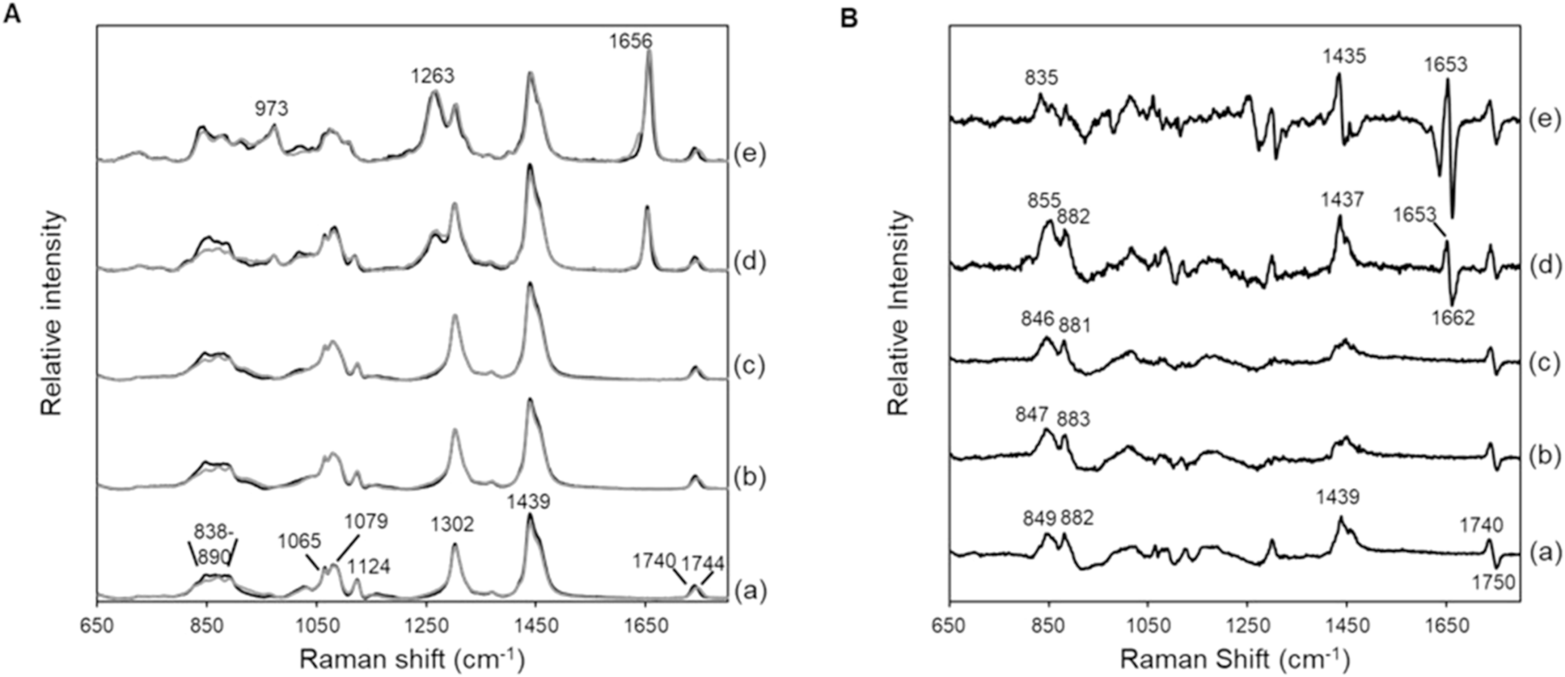
(A) Raman and (B) corresponding difference spectra of pure FAMEs and TAGs (shown in black and gray, respectively) containing (a) MA, (b) PA, (c) SA, (d) OA, and (e) LA fatty acyl groups. To facilitate comparisons, the FAME and TAG spectra are overlapped.

We developed a PCA-based simulation method to convert FAME spectra to TAG ones, enabling the application of FAME analytical models to TAG analysis. The differences between the spectra of FAMEs and TAGs bearing the same fatty acyl group were extracted using PCA, which is based on the least-squares method for multidimensional coordinate systems, uniquely determines scores and loadings, and is easier to apply than the simple difference spectral method. Δ*S*_D*i*_ can be obtained by subtracting the FAME and TAG spectra also. In the PCA of two data sets, PC1 indicates the difference between the data sets, and noise components are suppressed. Consequently, the simulated TAG spectrum is obtained using eqs 1 and ([Disp-formula eq2]).

Figure 3 shows the PCA score and loading plots of MAm and trimyristin, which contained MA acyl groups (the corresponding plots for PA, SA, OA, and LA are shown in Figure S3 in the Supporting Information). PC1 represents the major differences between the MAm and trimyristin spectra and is basically the same as the trimyristin and MAm difference spectrum in Figure 2B­(a).

**Figure 3:**
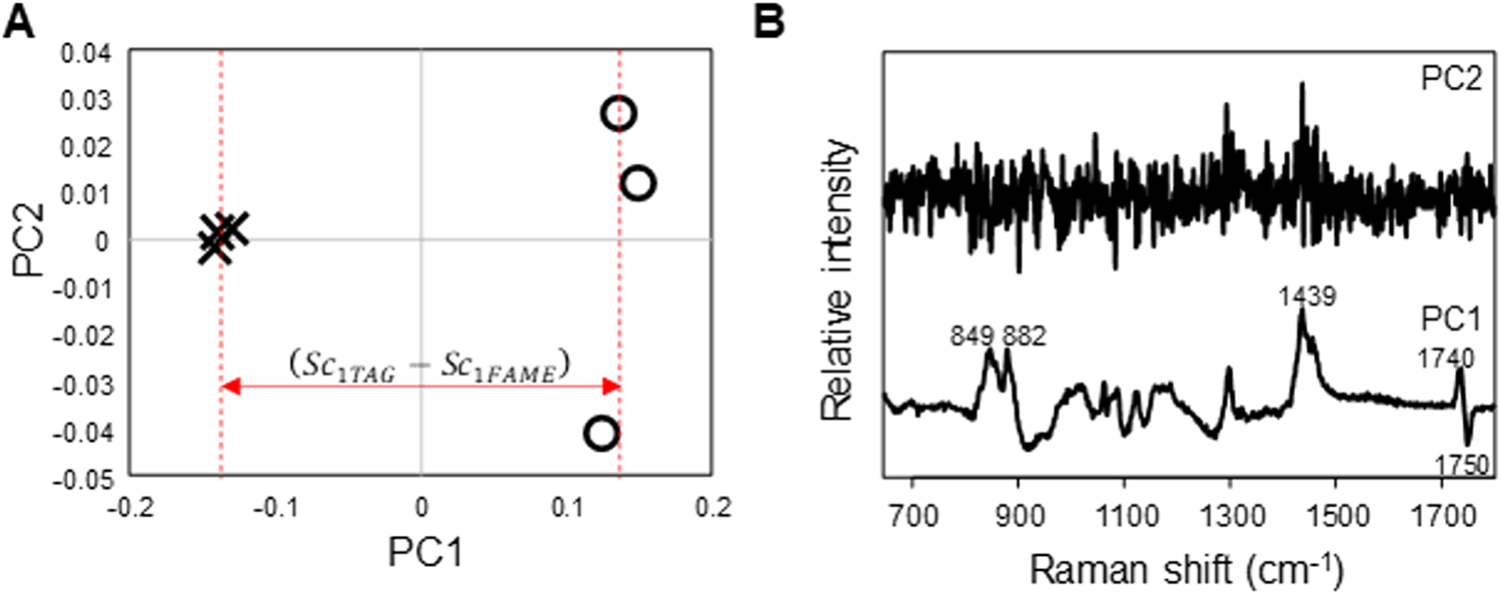
PCA (A) score and (B) loading plots of PC1 and PC2 of MA-acyl-groupcontaining FAMEs (○) and TAGs (×).

However, care must be taken because the difference spectrum depends on a subjective subtraction factor. In contrast, as PCA analysis automatically yields score values, the simulation spectra are obtained via eq 1 without any subjective processes. As the process of PCA can be described as least-squares methods, these equations are simply expressed as linear combinations. In particular, the spectral variance, Δ*S*_D*i*_, between each TAGi and FAMEi used to construct the simulation spectrum of the TAG mixture in eq 1 was obtained only for five TAGs that contain the same fatty acyl groups: trimyristin, tripalmitin, tristearin, triolein, and trilinolein. Since real fats contain TAGs with different fatty acyl groups linked via ester bonds in real fats, the current method may not be able to fully reproduce the TAG spectra, especially when the fatty acyl groups interact with each other. In this case, the evaluation will show low accuracy. A calibration data set for nonexistent TAG mixtures was generated by simulation according to eq 2 and used to construct the PLSR analytical model. For each combination of FAMEs and TAGs containing five different fatty acyl groups, the PCA results were similar. For TAGs and FAMEs containing MA, PA, SA, OA, and LA, PC1 explained 93%, 83%, 82%, 86%, and 85% of the total variances, respectively. In the loading plots shown in Figure 3B, spectral features indicate large differences, especially near the 1740 cm^–1^ band and in the 1500–1400 and 890–840 cm^–1^ regions, which were mainly attributed to C=O stretching mode, CH_2_/CH_3_ bending modes, and C–C and C–O stretching modes of hydrocarbon chain and ester groups, respectively. In contrast, PC2 mainly comprised white noise and baseline undulations. Figure 4 compares the spectra of PAm (a), simulated tripalmitin (b), and actual tripalmitin (c). It also shows the difference spectra between tripalmitin and PAm (d; c–a) and between tripalmitin and the simulated tripalmitin (e; c–b) on the same scale. Since no features were observed in the difference spectrum (e), the simulated spectrum accurately reproduces the actual spectrum.

**Figure 4:**
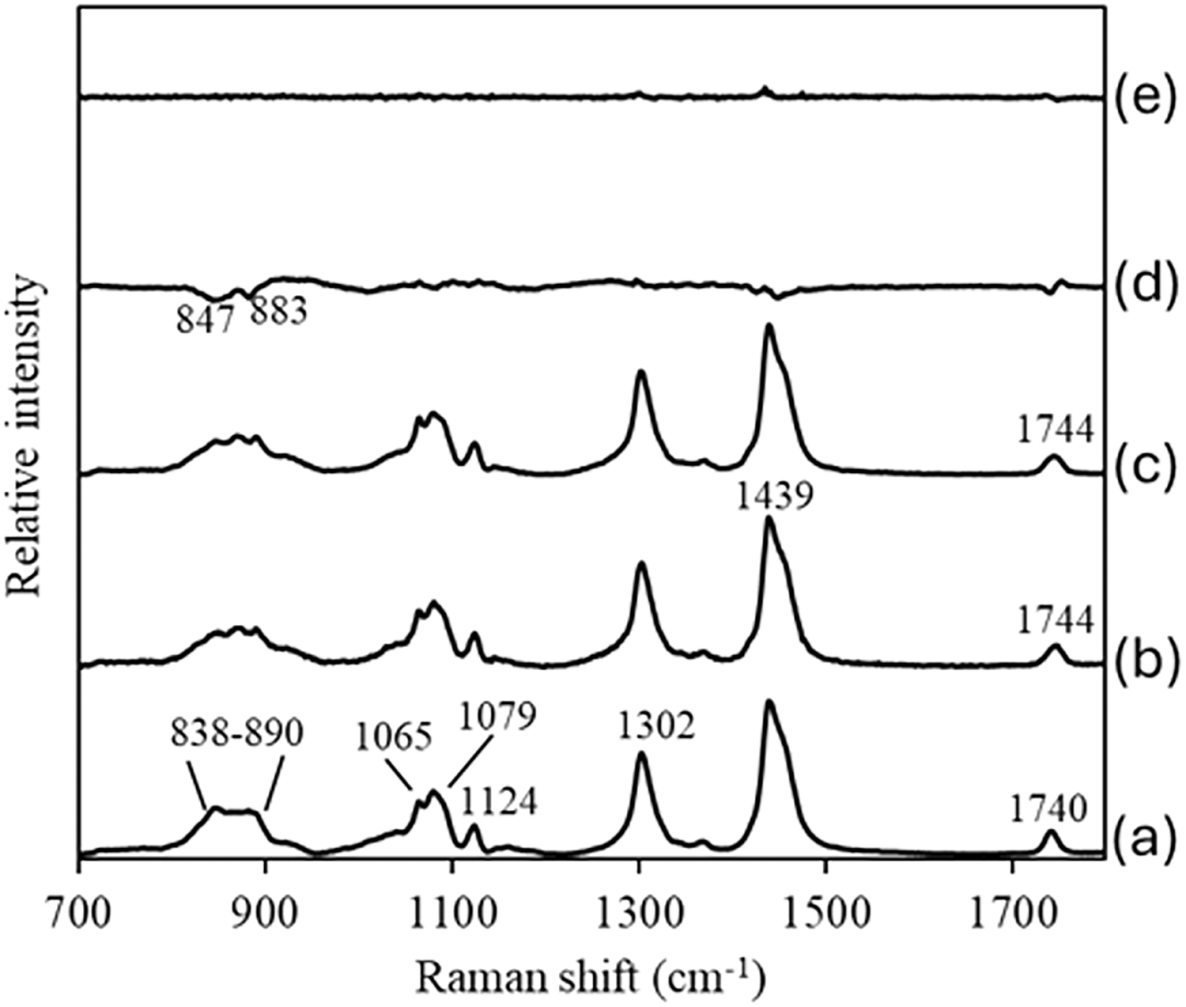
Raman spectra of PAm (a), simulated tripalmitin (b), tripalmitin (c) and their difference spectra (d) and (e). The difference spectra (d) and (e) show the difference between tripalmitin and PAm (c–a) and the difference between tripalmitin and the simulated tripalmitin (c–b), respectively, on the same scale.

The PLSR-based models constructed for TAGs bearing different fatty acyl groups were improved and trained by selecting the best windows in the wavenumber region (See Supporting Information for detailed explanation). The validation results of the optimized models are shown in Table 1. As shown in the leftmost RMSEP column, RMSEP was evaluated using the 20 TAG test data set made by simulation from the known concentrations of FAME mixtures. The models showed RMSEP values no higher than 5.7%. Of all the analytical models evaluated using the simulated TAG test data sets, the MA analytical model showed the lowest accuracy (5.7%). Regression coefficients for the five PLSR analytical models are shown in Figure 5. Values for areas excluded from the modeling scope are filled with “0” in the plots. Peaks near 1736 cm^–1^ assigned to the C=O stretching mode are observed in all the plots. This suggests that the ester bonds are sensitive to differences in the fatty acyl groups. Peaks around 1660 cm^–1^ originating from the C=C stretching modes are observed only in the plots of the unsaturated fatty acyl groups (d,e). In contrast, the regression coefficient plots of the saturated fatty acyl groups (a–c) exhibit complex bands in both positive and negative directions in the low frequency region, making it difficult to assign these peaks. The band region around 850 cm^–1^, where one of the most characteristic bands in the difference spectrum between tripalmitin and PAm (Figure 4(d)) is observed, is included in the regression coefficient plots of the MA, PA and SA models. These results suggest that the present method sufficiently simulates the TAG spectra to eliminate the difference between the methyl esters and glycerol esters.

**Figure 5:**
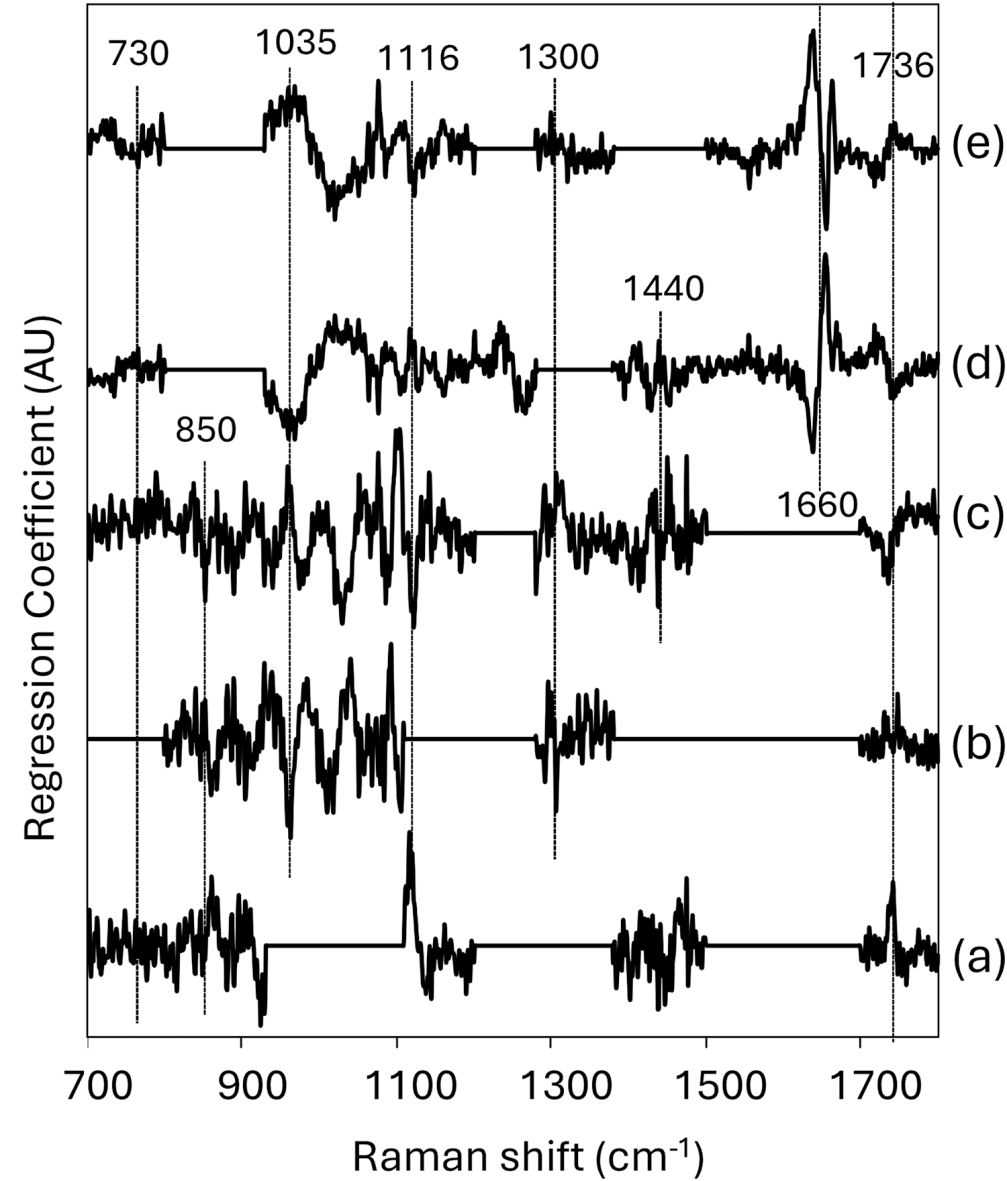
Regression coefficients of the simulated PLSR analytical models for trimyristin (a), tripalmitin (b), tristearin (c), triolein (d), and trilinolein (e).

**Table 1:** Optimized PLSR Analytical Models Built with Simulated Spectra for Trimyristin, Tripalmitin, Tristearin, Triolein, and Trilinolein, and Their Evaluation Results Using Olive and Sesame Oils^a^

					RMSEP		
fat prediction model	spectral window regions (cm^–1^)	*R*_C_^2^	*R*_P_^2^	RMSEC	simulated test samples	olive oil	sesame oil
myristate (14:0)	1800–1700, 1500–1380, 1200–1110, 930–650	0.98	0.88	3.7	5.7	5.6	6.5
palmitate (16:0)	1800–1700, 1380–1280, 1110–800	0.96	0.80	5.0	4.6	3.7	4.8
stearate (18:0)	1800–1700, 1500–1280, 1200–650	0.96	0.93	5.4	4.3	2.5	4.1
oleate (18:1)	1800–1380, 1280–930, 800–650	0.99	0.99	2.4	2.4	4.6	4.6
linoleate (18:2)	1800–1500, 1380–1280, 1200–930, 800–650	0.99	0.97	1.4	2.1	2.4	4.4

a *R*_C_^2^ = *R* ^2^ of calibration; *R*_P_^2^ = *R* ^2^ of prediction.

### Evaluation of the Analytical Model for the Simulated TAGs Spectra Using Edible Oils

The PLSR-based models were evaluated three times each using olive and sesame oils. The fatty acyl group compositions (expressed as mole percentages) of the oils, as analyzed using GC and PLSR, are shown in Table 2. Here, the standard deviations (SDs; μ) shown alongside the mean value represents the deviation between Raman analyses, obtained by the following equation:

$$\mu =\sqrt{\frac{\sum_{i=1}^{n}{\left({ŷ}_{i}-\mathrm{y̅}\right)}^{2}}{n-1}}$$

Here, “*y̅*” is the mean of the measured values “*ŷ*_*i*_” (*y̅* = ∑_*i*=1_^*n*^ *y*_*^i*_/*n*). The analysis was repeated three times, and the SD indicates the dispersion around the mean value of the triplicate data points. In contrast, the RMSEP of edible oils in Table 1 indicates the deviation between the Raman- PLSR and GC results. The fatty acyl group concentration determined by GC was used as the actual concentration “*y*”. Thus, the SD and RMSEP represent precision and accuracy of the Raman- PLSR analyses, respectively. For the edible oils, the RMSEP values were similar to those obtained for the simulated test samples. This indicates that the errors in Raman–PLSR measurements were primarily due to noise in the Raman spectra rather than irregularities in the simulation. Thus, the analytical model based on the simulated TAG spectra functioned properly. Improving the quality of the Raman spectra can lead to more accurate PLSR models. However, if the simulated TAG model did not fit experimentally measured TAG spectra, the SDs of the real samples would be much larger than those of the same simulated test data set. This is not the case.

**Table 2:** FA Compositions Measured by GC and Raman Spectroscopy for Olive Oil, Sesame Oil, and LD in Adipocytes^a^

	content ± stdev (%)					
	olive		sesame		LDs in adipocytes	
fatty acid	GC-FID	Raman	GC-FID	Raman	GC-FID	Raman
C14:0	n.d.	4.83 ± 1.09	0.03 ± 0.01	1.76 ± 2.04	3.77 ± 0.22	14.33 ± 7.85
C16:0	10.68 ± 0.17	7.62 ± 1.17	6.96 ± 0.35	5.19 ± 2.11	44.80 ± 1.15	47.73 ± 7.77
C18:0	2.48 ± 0.02	3.18 ± 1.75	3.76 ± 0.02	2.65 ± 1.41	10.52 ± 1.63	2.42 ± 2.79
C18:1(n-7 and n-9)	78.27 ± 0.08	73.54 ± 0.44	49.84 ± 0.15	51.96 ± 1.93	16.68 ± 1.65	21.65 ± 4.93
C18:2 (n-6)	6.19 ± 0.01	3.85 ± 0.14	33.89 ± 0.04	29.65 ± 0.92	1.71 ± 0.15	6.39 ± 3.28
C10:0	n.d.		n.d.		0.06 ± 0.08	
C12:0	n.d.		n.d.		0.34 ± 0.09	
C14:1 (n-5)	n.d.		n.d.		0.20 ± 0.08	
C16:1(n-7)	0.50 ± 0.01		0.14 ± 0.01		6.53 ± 0.04	
C17:0	0.05 ± 0.00		0.05 ± 0.01		0.29 ± 0.12	
C17:1(n-8)	n.d.		n.d.		0.39 ± 0.12	
C18:3(n-3)	n.d.		n.d.		0.05 ± 0.07	
C18:3 (n-6)	0.62 ± 0.00		3.41 ± 0.01		n.d.	
C20:0	0.46 ± 0.01		0.57 ± 0.04		0.22 ± 0.05	
C20:1(n-9)	0.36 ± 0.02		0.39 ± 0.31		0.08 ± 0.13	
C20:2(n-9)	n.d.		0.04 ± 0.00		0.31 ± 0.03	
C20:3(n-6)	n.d.		n.d.		0.19 ± 0.19	
C20:4(n-6)	n.d.		n.d.		2.92 ± 0.66	
C20:5(n-3)	n.d.		n.d.		0.21 ± 0.03	
C22:0	0.02 ± 0.00		n.d.		0.35 ± 0.03	
C22:1(n-9)	n.d.		n.d.		0.12 ± 0.17	
C22:6(n-3)	n.d.		n.d.		1.87 ± 0.34	
C24:0	n.d.		n.d.		0.80 ± 0.14	
C24:1(n-9)	n.d.		n.d.		0.20 ± 0.18	
unknown	0.37	6.98	0.92	8.79	7.39	7.48
Total	100	100	100	100	100	100

a n.d. = not detected.

The total contents of the five fatty acyl groups analyzed using GC were 97.3% and 93.0% for olive and sesame oils, respectively. The same measured using the combined Raman spectroscopy and PLSR models were 93.0% and 91.2%, respectively, similar to those measured using only GC. In the olive and sesame oils, the OA concentrations were 77.98% ± 0.08% and 48.33% ± 0.15% (as measured using GC) and 73.54% ± 0.44% and 51.96% ± 1.93% (as measured using Raman spectroscopy), respectively. The mean OA concentrations measured using GC and Raman spectroscopy were similar, only differing by approximately 4%. Additionally, Table 1 shows RMSEP values, suggesting that the Raman–PLSR analysis accurately predicted high concentrations of FAs. In the olive and sesame oils, the MA concentrations were not detected and 0.03% ± 0.01% (as measured using GC) and 4.83% ± 1.09% and 1.76% ± 2.04% (as measured using Raman spectroscopy), respectively. As the RMSEP values in Table 1 indicated that 5%–6% error can be expected for the Raman–PLSR analysis of MA, Raman–PLSR is likely inadequate for measuring low concentrations of FAs.

### Quantitative Analysis of FA Compositions in Live Cells

In this study, the models trained using simulated spectra for quantitatively analyzing fatty acyl groups in TAG samples were applied to estimate the FA compositions of LDs in live cultured mouse adipocytes. The adipocyte fatty acyl group compositions analyzed using GC and Raman–PLSR are compared in Table 2. For GC analyses, fat samples were extracted from each dish of cultured adipocytes and transmethylated to FAMEs. For the GC-analyzed LDs, the fatty acyl group concentrations represent the means and SDs for five replicates rather than individual LDs. The total fatty acyl group concentration measured using GC was 77.48%, comprising MA (14:0; 3.77%), PA (16:0; 44.80%), SA (18:0; 10.52%), OA (18:1, n-9 and n-7; 16.68%), and LA (18:2, n-6; 1.71%). Because the Raman spectra of vaccenic acid and OA were very similar, vaccenic acid (18:1, n-7) was counted as OA, although they had been analyzed separately by GC. Except for these fatty acyl groups, the most abundant components were palmitoleic (16:1; 6.53%), arachidonic (20:4; 2.92%), and docosahexaenoic (22:6; 1.87%) acids, which were more abundant than LA. The fatty acyl group compositions in adipocyte LDs (as estimated using Raman spectroscopy) are shown in the rightmost column in Table 2. Raman spectra were recorded for 30 LDs in each of the five dishes, for a total of 150 spectra. For the Raman–PLSR data, the SDs indicated the data variance for individual LDs. In the Raman–PLSR measurements, the SDs obtained for LDs were larger than those obtained for the edible oils, suggesting greater variation among individual LDs in live adipocytes. As Raman–PLSR analysis was directly applied to individual LDs in untreated samples, these data indicated the variance in fatty acyl chain compositions.

Live cultured adipocytes in untreated samples were nondestructively analyzed using Raman spectroscopy. Figure 6A shows a bright-field image of live adipocytes, which cytoplasms contained variously sized single or multiple LDs. The laser was focused on the center of an LD. Raman spectra of the LDs in the cells are shown in Figure 6B. The band at 1744 cm^–1^ was assigned to the C=O stretching mode, suggesting that FAs were stored as acyl groups in TAGs. The large variations among the bands at 1260 and 1656 cm^–1^ suggested that the unsaturated fatty acyl group concentrations substantially fluctuated. Figure 6C compares LDs’ fatty acyl group compositions measured using Raman–PLSR and GC. Although the overall compositions were similar, the corresponding SDs were notably different. In the Raman–PLSR analysis (Figure 6C), error bars represent the SDs calculated for five averages measured for 30 cells in each dish and are shown for comparison with the corresponding GC measurements. As shown in Table 2, the GC measurements showed a larger SD for LDs than edible oils, possibly because fat extraction and methylation may have changed the fatty acyl group composition (as the cellular suspension contained traces of fats and various substances, such as many enzymes) or because of the variability among the culture dishes. As cells sometimes affected each other in a wide range of manners and globally affected the same dish, each culture dish acquired different properties. However, the origin of the distribution was difficult to elucidate based on only GC measurements. For the Raman–PLSR measurements, the MA, PA, SA, OA, and LA SDs were 3.47, 3.43, 0.99, 1.32, and 1.53, larger than the standard errors expected for 30 cells ( $\mu_{a}=\mu /\sqrt{30}$ ; 1.43, 1.42, 0.51, 0.90, and 0.60), respectively. Hence, the Raman–PLSR measurements support the variability among the culture dishes as the origin of the distribution.

**Figure 6:**
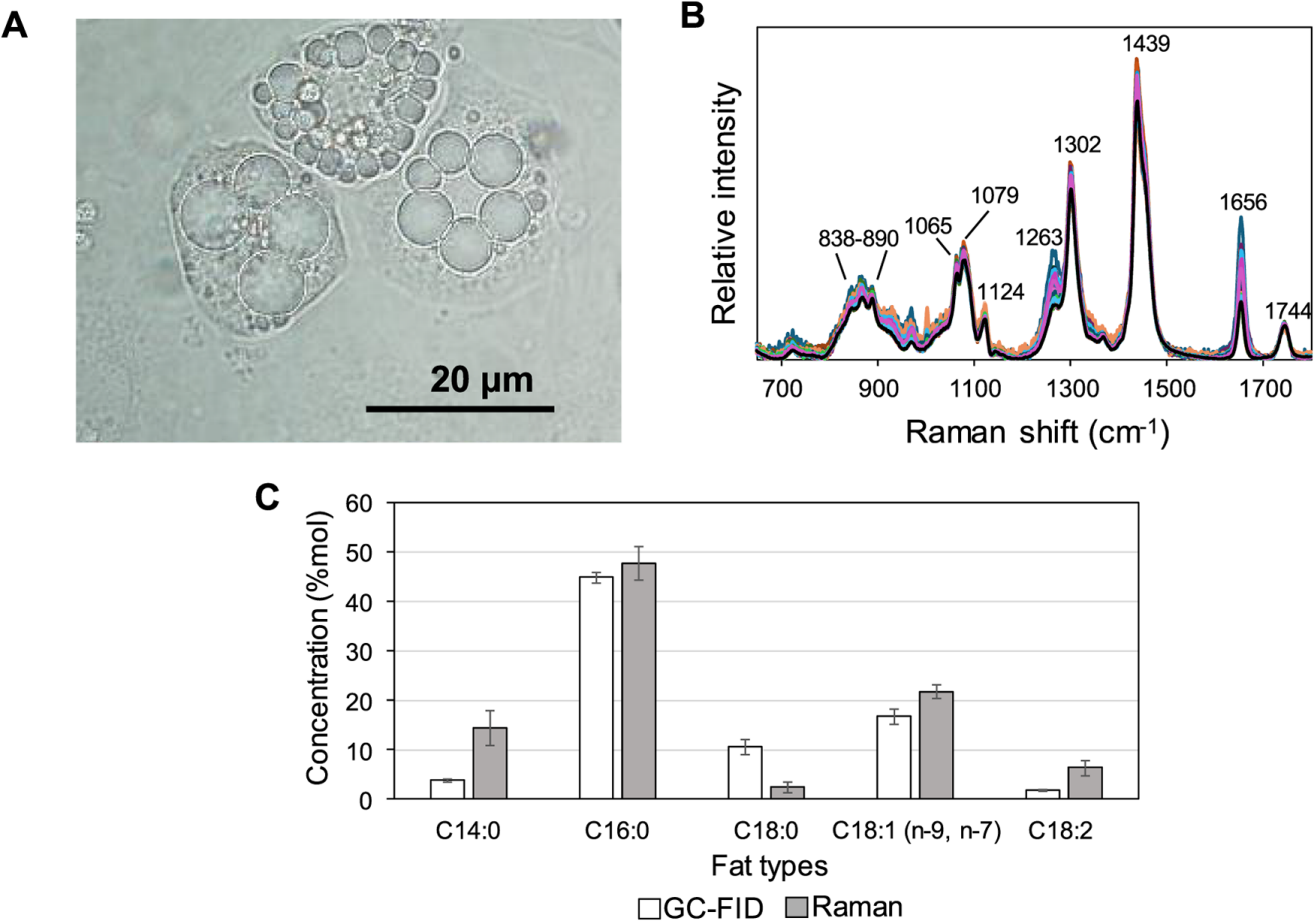
A bright field image of adipocyte (A), Raman spectra of LDs in the cells (B) and composition of FAs (C) estimated by GC and Raman analyses. Error bars indicate the standard errors of five replicates.

We investigated whether LDs exhibited cell-specific characteristics. According to the HCA results of the Raman–PLSR-predicted fatty acyl group compositions of 50 LDs in cultured adipocytes (the data are shown in Figure S4 of the Supporting Information), compared to the overall variation, the variation among the LDs was relatively small for individual cells, suggesting that in LDs, the fatty acyl group composition exhibited cell-specific heterogeneity. Because Raman–PLSR analysis is a direct sampling-free method, it is free from any error generated during sample treatment. These results suggested that Raman spectroscopy combined with chemometrics has excellent potential for quantitatively analyzing FA compositions in localized and traces of fats, such as LDs in live cells.

## Conclusions

This study demonstrated the feasibility of combining Raman spectroscopy with chemometrics for practical application in rapidly and nondestructively quantitatively analyzing the fatty acyl group compositions of live cells and edible oils. The proposed technique enables the construction of analytical models for samples for which pure components are unavailable. PLSR analytical models constructed based on TAG spectra simulated using FAME spectra were applied for analyzing fatty acyl group compositions of fats, generating relatively small errors (<6.5%). The proposed technique was applied to measure the fatty acyl group compositions of individual LDs in live adipocytes without pretreating any samples. Because Raman spectroscopy does not require any sample pretreatments, which may generate additional error in FA analysis, it can be used to measure native fatty acyl group concentrations in live cells and tissues. Additionally, the results suggested that fatty acyl group compositions substantially varied among adipocytes. Although the proposed technique holds the potential to usher in a new era of FA metabolism research, several accuracy issues must first be resolved. Furthermore, the noninvasiveness of Raman spectroscopy enables the application of the proposed technique in human healthcare.

## Supplementary Material



## Notes

CLSR, NNLSR, and PLSR analyses, fat extraction and preparation for GC–FID analysis, additional PCA score plots for Raman spectra of FAME and TAG, HCA dendrogram, table of Raman band assignments, table of evaluation results for quantitative analyses of FAME mixtures with PLSR, CLSR, and NNLSR, and table of results of FA composition prediction of edible oils using Raman–PLSR models built for FAMEs (PDF)

## Abbreviations

FA: fatty acid
TAG: triacylglycerol
FAME: fatty acid methyl ester
PLSR: partial least-squares regression
GC: gas chromatography
FID: flame-ionized detector
CM: chylomicron
BHRP: ball lens top hollow optical fiber Raman probe
LA: linoleic acid
PA: palmitic acid
SA: stearic acid
OA: oleic acid
LD: lipid droplets
CLSR: classical least-squares
NNLSR: non-negative least-squares
PCA: principal component analysis
BHT: butyl hydroxy toluene
FBS: fetal bovine serum
IBMX: isobutylmethylxanthine
MAm: methyl-myristate
PAm: methyl-palmitate
SAm: methyl-stearate
OAm: methyl-oleate
LAm: methyl-linoleate
NA: numerical aperture
EDTA: ethylenediaminetetraacetic acid
SPE: solid phase extraction
PC: principal component
HCA: hierarchical clustering analysis
RMSE: root mean squared error
