# Supplementary material for: Raman Spectroscopy–Based Quantitative Analysis of Fatty Acid Compositions of Lipid Droplets in Live Cells
Source: Anal Chem. 2026 Mar 9;98(11):8023–33. doi: 10.1021/acs.analchem.5c04227 (PMC13019424; doi:10.1021/acs.analchem.5c04227)
Supplement: Supplementary file 1 [file ac5c04227_si_001.pdf]

Supporting Information

Raman Spectroscopy–Based Quantitative Analysis of Fatty Acid Compositions of Lipid Droplets in Live Cells

*Pradjna N. Paramitha[1], Keita Iwasaki[1,2], Bibin B. Andriana[2], Yurika Otoki[3], Ibuki Kusumoto[3], Yukihiro Ozaki[2], Kiyotaka Nakagawa[3], Hidetoshi Sato[*1,2]*

[1]Graduate School of Science and Technology, Kwansei Gakuin University, 1 Gakuen Uegahara, Sanda, Hyogo 669-1330, Japan

[2]School of Biological and Environmental Sciences, Kwansei Gakuin University, 1 Gakuen Uegahara, Sanda, Hyogo 669-1330, Japan

[3]Food Function Analysis Laboratory, Graduate School of Agricultural Science, Tohoku University, Sendai, Miyagi 980-8572, Japan

[*]Corresponding author.

E-mail address: hidesato@kwansei.ac.jp (H. Sato)

Tel.: +81-79-565-7228; Fax: +81-79-565-7228

Contents

CLSR, NNLSR, and PLSR analyses (**Figure S1**, **Figure S2**)--S3

Fat extraction and preparation for GC–FID analysis--S7

Figure S3. PCA score and loading plots of FAME and TAG Raman spectra-----------------------S9

Figure S4. LD hierarchical clustering plot--S10

Table S1. Raman band assignments---S11

Table S2. Evaluation results of PLSR, CLSR, and NNLSR analyses-------------------------------S12

Table S3. FA compositions prediction in edible oils by FAME models-----------------------------S13

CLSR, NNLSR, and PLSR analyses

Classical least squares regression (CLSR)

CLSR is a direct regression method that requires full knowledge of all the training samples' components.[1,2] Generally, CLSR is applied to simultaneously estimate the compositions of all the components. The following formula is applied in CLSR:

$$\mathbf{A} = \mathbf{CK} + \mathbf{E}$$

A = spectra matrix

C = component quantitative value (concentration)

K = spectral intensities of the components matrix

E = noise matrix

Nonnegative least squares regression (NNLSR)

NNLSR is similar to CLSR, but it restricts the coefficient vector (C) to always be positive,[3] which is reasonable because concentrations are nonnegative quantities.

Partial least squares regression (PLSR)

PLSR is an indirect regression method that only requires knowledge of the concentrations of substances of interest. Five individual PLSR models were constructed to predict the concentration of each FAME or fatty acyl group. To construct the proposed analytical model, covariances between independent and dependent variables were maximized.[4]

PLSR model building

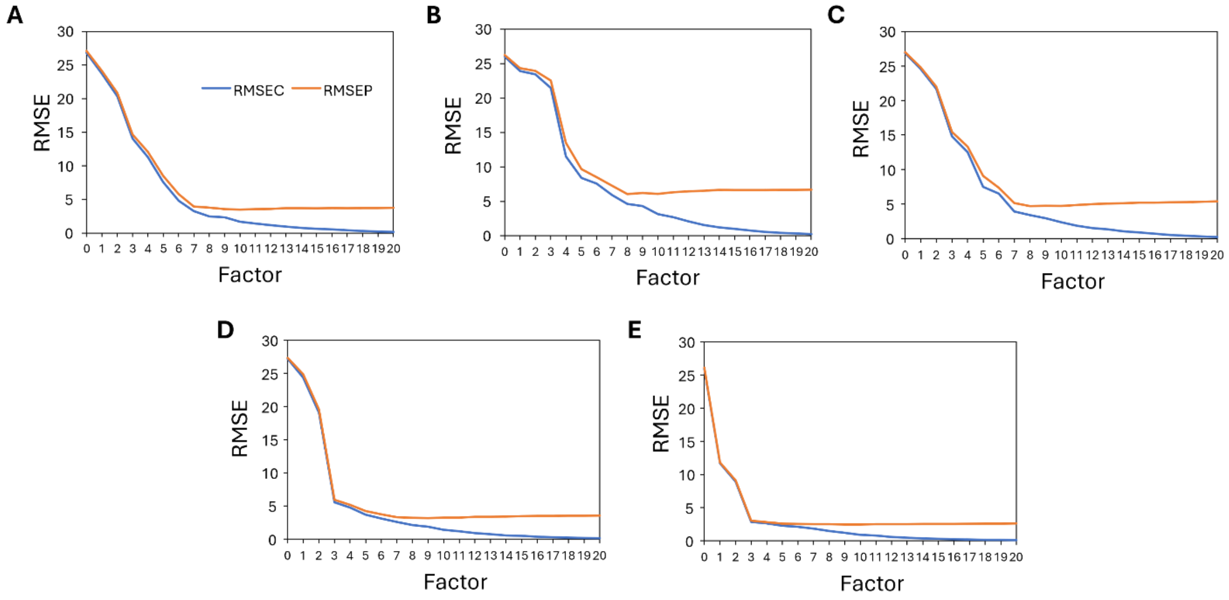

Figure S1. Plots of RMSE vs number of factors used in PLSR models built for FAMEs, i.e., MAm (A), PAm (B), SAm (C), OAm (D), and LAm (E).

The PLSR prediction models built for MAm, PAm, SAm, OAm, and LAm were constructed based on 7, 8, 7, 5, and 3 factors, respectively. **Figure S1** shows the relationship between RMSE and factor numbers for these PLSR models. When the factor number is small, the RMSEC and RMSEP decrease together. However, when the factor number is greater than eight (Figure S1A), the RMSEP slightly increases despite the decrease in RMSEC. In this case, the calibration model is overfitted when the factor number is greater than eight and the model is optimized with seven factors. The PLSR prediction models built for simulated trimyristin, tripalmitin, tristearin, triolein, and trilinolein were constructed based on 7, 7, 6, 6, and 6 factors, respectively.

Moving window technique for improvement of PLSR models

Each PLSR model was trained to improve the accuracy for predicting specific FAMEs or TAGs by using a modified backward interval PLS (biPLS) method. BiPLS is a method to select the spectral regions that produce the best analytical model in PLS analysis.[5,6] Initially, a PLS model is

constructed using the full spectrum range. The spectrum is then segmented into n subintervals. New PLS models were then built by removing one subinterval at a time until the best analytical model with the lowest RMSEC and RMSEP is obtained. In this study, BiPLS with modifications was applied to select the spectral regions of each analytical model. Generally, the subintervals of the spectrum are determined by dividing the full spectrum range into n subintervals having equal length. In this study, the subintervals were determined by dividing the full spectrum range into several visible peaks in the Raman spectrum of fat: 650-800, 800-930, 930-1000, 1000-1050, 1050-1110, 1110-1200, 1200-1280, 1280-1380, 1380-1400, 1400-1500, 1500-1600, 1600-1700, 1700-1800 cm^{-1}, as shown in the spectra of triolein in **Figure S2**. The highest RMSEP values obtained using the PLSR models for FAMEs constructed based on the 1700–650 and 1500–650 cm^{-1} regions were 6.9 and 6.4, suggesting that the latter model possessed a higher prediction accuracy. These results showed that the selection of specific spectral windows as predictors enhanced the prediction accuracies of certain analytical models. For each FAME, the optimal PLSR model possessed the lowest RMSEC and RMSEP values. The RMSEC and RMSEP values were similar, suggesting that the PLSR models did not overfit the data. When the model was overfitted, the difference between the RMSEC and RMSEP values increased, although the RMSEC value improved.

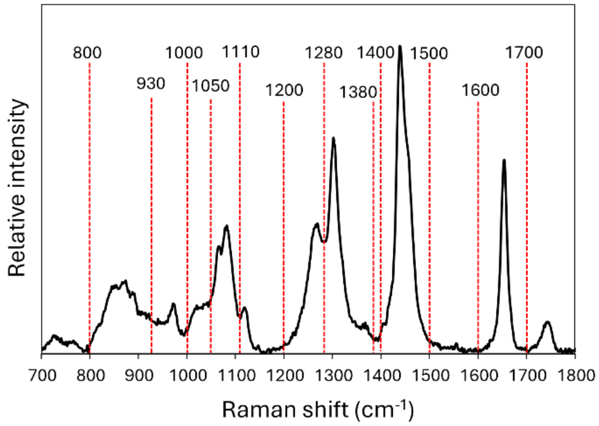

Figure S2. Subintervals applied for searching the best spectral window using biPLS depicted in Raman spectra of triolein.

References

(1) Galeano Díaz, T.; Guiberteau, A.; Ortíz Burguillos, J. M.; Salinas, F. Comparison of Chemometric Methods: Derivative Ratio Spectra and Multivariate Methods (CLS, PCR and PLS) for the Resolution of Ternary Mixtures of the Pesticides Carbofuran Carbaryl and Phenamifos After Their Extraction into Chloroform. *Analyst*. **1997**, 122(6), 513–517.

(2) Zhou, Y.; Cao, H. An Augmented Classical Least Squares Method for Quantitative Raman Spectral Analysis against Component Information Loss. *Sci. World J*. **2013**, 2013(1), 306937.

(3) Slawski, M.; Hein, M. Non-Negative Least Squares for High-Dimensional Linear Models: Consistency and Sparse Recovery without Regularization. *Electron. J. Statist.* **2013**, 7, 3004-3056.

(4) Wold, S.; Sjöström, M.; Eriksson, L. PLS-Regression: A Basic Tool of Chemometrics. *Chemom. Intell. Lab. Syst.* **2001**, 58(2), 109–130.

(5) Ran, Z.; Sun, L.; Liu, Y.; Pan, X.; Li, J.; Liu, Y. Forward and backward interval partial least squares method for quantitative analysis of frying oil quality. *Infrared Phys. Technol*. **2020**, 105, 103207.

(6) Ma, C.; Zhai, L.; Ding, J.; Liu, Y.; Hu, S.; Zhang, T.; Tang, H.; Li, H. Raman spectroscopy combined with partial least squares (PLS) based on hybrid spectral preprocessing and backward interval PLS (biPLS) for quantitative analysis of four PAHs in oil sludge. *Spectrochim. Acta A Mol. Biomol. Spectrosc*. **2024**, 310, 123953.

Fat Extraction and Preparation for GC–FID Analysis.

Frozen mouse adipocyte–differentiated cells were thawed and sonicated for 5 min. A modified Folch method was applied to extract the total lipids from cell suspensions.[1-3] Briefly, to 1 mL of a cell suspension, 0.2 mL of 0.9% potassium chloride containing 1 mM ethylenediaminetetraacetic acid (EDTA) and 4.8 mL of a chloroform/methanol 2:1 (v/v) mixture were added and vortexed for 5 min. The mixture was centrifuged at 3,000 rpm for 20 min at 4°C. The total lipids were collected from the bottom layer (cells firmly attached on the dish bottom; they do not include floated cells), while 2.8 mL of a chloroform/methanol 10:1 (v/v) mixture was added to the remaining top layer, which was vortexed again for 5 min and centrifuged at 3,000 rpm for 20 min at 4°C, and the bottom layer was collected and added to the previously collected bottom layer. The solvent was evaporated using a centrifugal evaporator at 1500 rpm and 30°C. Prior to solid-phase extraction (SPE) to separate the neutral and polar lipids, the total lipid extract was reconstituted in 0.5 mL of a chloroform/isopropanol 2:1 (v/v) mixture. A Strata SI-1 silica column (100 mg, 1 mL) was used for SPE. The column was equilibrated by loading it with 1.5 mL each of methanol and a chloroform/isopropanol 2:1 (v/v) mixture. Then, the total lipids and 1.5 mL of a chloroform/isopropanol 2:1 (v/v) mixture were added to the column, subsequently. Next, the column was flushed using 1.5 mL of a chloroform/isopropanol 2:1 (v/v) mixture, and the fraction was collected as a neutral lipid. The solvent was evaporated from the neutral lipid fraction using a centrifugal evaporator at 1500 rpm and 30°C, and the neutral lipid extract was stored at −80°C until transmethylation.

The neutral lipid extract was subjected to acid-catalyzed transmethylation, as described in Lepage and Roy (1986).[4] The same method was used for transmethylation of olive oil and sesame oil (100 μL each). Briefly, 1.5 mL of an acetyl chloride/methanol/benzene 1:8:2 (v/v) mixture was added to a screw-capped tube containing the neutral lipid extract, and the mixture was vortexed and

transesterified at 100°C for 1 h and subsequently cooled to room temperature. For neutralization, 5 mL of 6% aqueous potassium carbonate was added to the tube and mixed. The FAMEs were extracted from the mixture by adding 1 mL of hexane to the reaction tube and mixing. The top layer was left to separate from the bottom aqueous layer for 30 min and then collected in a separate tube. The extraction was repeated once by adding 1 mL of hexane to the remaining bottom layer. Then, the top layer was collected in the same tube as that of the previously collected top layer. For GC–FID analysis, the solvent was evaporated under a nitrogen stream to prevent oxidation, and the solid residue was redissolved in 100 μL of hexane.

References

(1) Folch, J.; Lees, M.; Stanley, G. H. S. A SIMPLE METHOD FOR THE ISOLATION AND PURIFICATION OF TOTAL LIPIDES FROM ANIMAL TISSUES. *J. Biol. Chem.* **1957**, 226(1), 497–509.

(2) Kato, S.; Nakagawa, K.; Suzuki, Y.; Asai, A.; Nagao, M.; Nagashima, K.; Oikawa, S.; Miyazawa, T. Liquid Chromatography–Tandem Mass Spectrometry Determination of Human Plasma 1-Palmitoyl-2-Hydroperoxyoctadecadienoyl-Phosphatidylcholine Isomers via Promotion of Sodium Adduct Formation. *Anal. Biochem.* **2015**, 471, 51–60.

(3) Saito, H.; Kato, S.; Shimizu, N.; Takahashi, T.; Jutanom, M.; Ito, J.; Kasatani, S.; Nakagawa, K. LC-MS/MS Analysis of Milk Triacylglycerol Hydroperoxide Isomers Which Are Generated Corresponding to the Photo- and Thermal-Oxidation. *Food Res. Int.* **2024**, 178, 113913.

(4) Lepage, G.; Roy, C. C. Direct Transesterification of All Classes of Lipids in a One-Step Reaction. *J. Lipid Res*. **1986**, 27(1), 114–120.

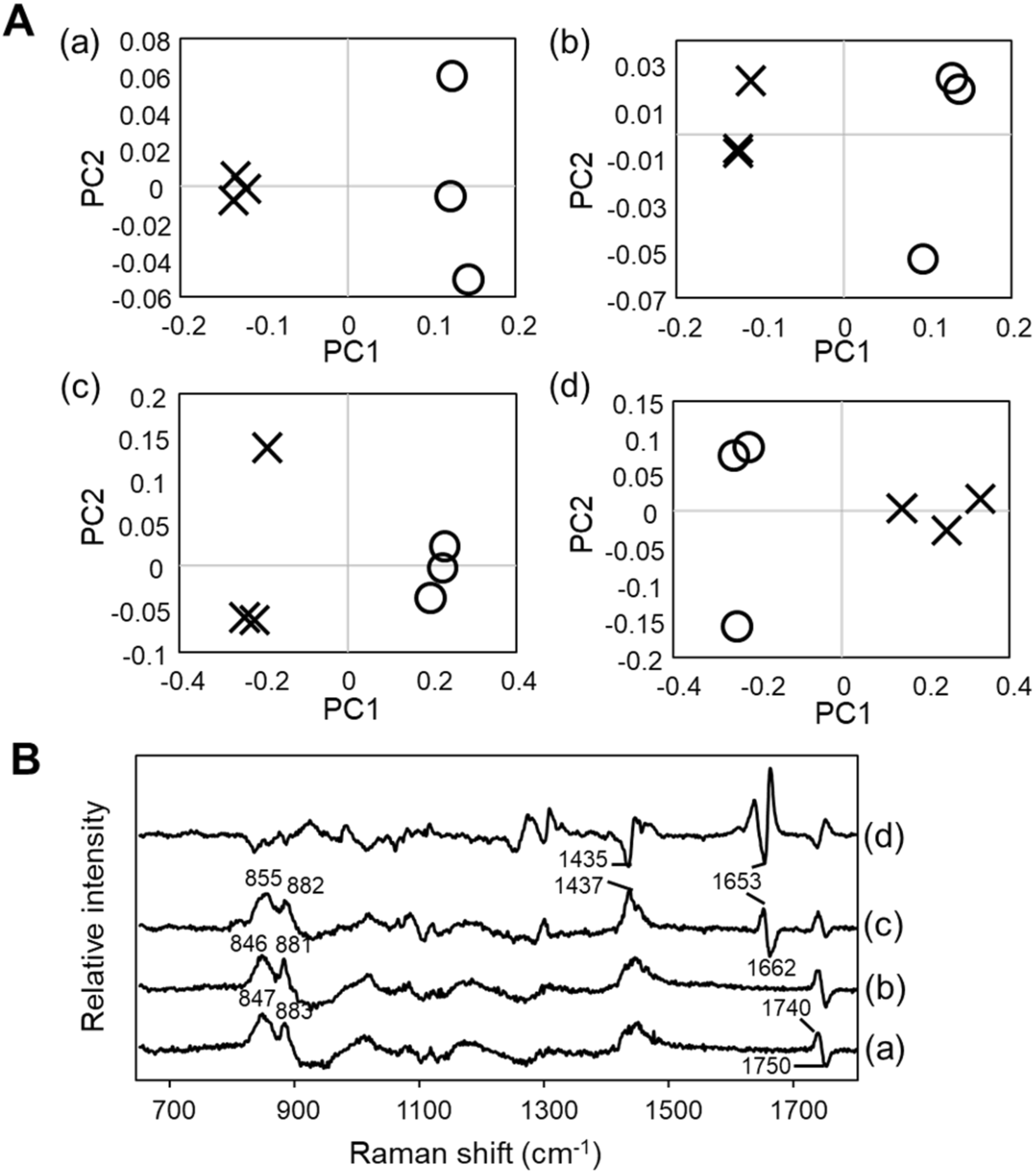

Figure S3. (A) PC1 and PC2 PCA score and (B) loading plot of PC1 constructed based on Raman spectra of FAMEs (◯) and TAGs (×) containing (a) PA, (b) SA, (c) OA, or (d) LA fatty acyl groups.

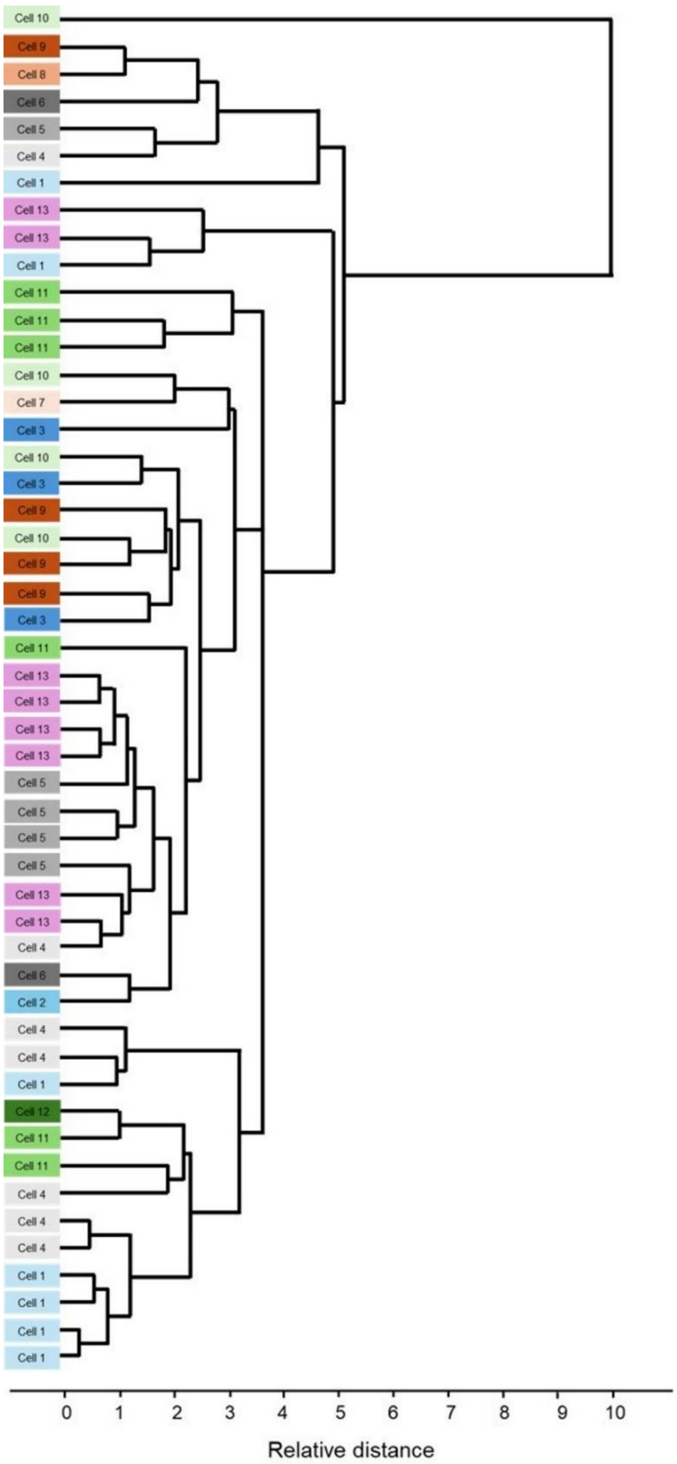

Figure S4. Hierarchical clustering plot of LDs in thirteen live adipocytes. Euclidean-distance-based medium linkage clustering was applied to the LDs' predicted fatty acyl group compositions. The data in each cell are classified using different colors.

Table S1. Typical Raman band assignments for fats.

Raman band (cm^{-1})	Assignment
1744	C=O stretching of TAGs
1740	C=O stretching of FAMEs
1656	$(C=C)_{cis}$ stretching
1500-1400	CH_2/CH_3 bending
1305-1295	CH_2 twisting
1270-1260	C=C-H bending
1050-1200	C-C, C-O-C stretching
970	H-C=C-H out-of-plane bending
900-820	CH_2 rocking, C-O stretching

Table S2. Evaluation results for FAME mixtures quantitatively analyzed using PLSR, CLSR, and NNLSR.

Fat prediction model	Spectral window regions (cm^{-1})	PLSR				CLSR		NNLSR	
		R^2_C	R^2_P	RMSEC	RMSEP	R^2_P	RMSEP	R^2_P	RMSEP
Methyl-myristate (14:0)	1700-650	0.98	0.98	3.3	3.9	0.71	28.9	0.34	20.9
Methyl-palmitate (16:0)	1700-650	0.97	0.95	4.7	6.9	0.47	48.5	0.08	19.5
	1500-650	0.97	0.94	4.3	6.4	0.48	43.2	0.08	19.5
Methyl-stearate (18:0)	1700-650	0.98	0.96	3.9	5.1	0.28	65.6	0.07	32.5
	1500-650	0.98	0.97	3.3	4.7	0.39	50.9	0.05	21.7
Methyl-oleate (18:1)	1700-650	0.98	0.98	3.7	4.4	0.61	12.5	0.54	15.0
	1700-1400, 1280-650	0.98	0.98	3.5	3.9	0.63	13.4	0.59	15.3
Methyl-linoleate (18:2)	1700-650	0.99	0.99	2.9	2.4	0.85	4.6	0.90	4.4

*R^2_C = R^2 of calibration; R^2_P = R^2 of prediction

Table S3. Fatty acyl group compositions of edible oils, as predicted using PLSR models constructed with FAME datasets.

Fat prediction model	Spectral window regions (cm^{-1})	R^2_C	R^2_P	Olive oil: Predicted content ± stdev (%)	Olive oil: RMSEP	Sesame oil: Predicted content ± stdev (%)	Sesame oil: RMSEP
Myristate (14:0)	1700-1500, 1400-930, 800-650	0.97	0.67	-13.26 ± 1.24	13.3	-5.20 ± 1.65	5.4
Palmitate (16:0)	1400-930, 800-650	0.93	0.93	34.78 ± 1.74	24.1	26.11 ± 2.46	19.2
Stearate (18:0)	1400-930, 800-650	0.95	0.47	8.88 ± 1.90	6.6	12.40 ± 2.82	8.9
Oleate (18:1)	1700-1500, 1280-930, 800-650	0.97	0.99	49.52 ± 0.60	28.5	8.94 ± 1.92	39.4
Linoleate (18:2)	1700-1500, 1400-930, 800-650	0.99	0.99	22.05 ± 0.42	15.9	54.66 ± 0.59	20.8

*Spectral regions which differ between FAMEs and TAGs, i.e., 1500-1400 and 930-800 cm^{-1}, were excluded for analysis.
$R^2_C = R^2$ of calibration; $R^2_P = R^2$ of prediction.
